# Sympathetic overactivation and catecholamine toxicity: mechanisms and therapeutic strategies for neurogenic heart injury following acute ischemic stroke

**DOI:** 10.3389/fcvm.2025.1632704

**Published:** 2025-10-10

**Authors:** Wang Guo, Hong-yu Li, Hong-xin Li, Qi-wen Nie, Zhi-hao Wang, Jian-hui Li, Qiang Tang

**Affiliations:** ^1^ Heilongjiang University of Chinese Medicine, Harbin, China; ^2^ The Second Affiliated Hospital of Heilongjiang University of Chinese Medicine, Harbin, China

**Keywords:** acute ischemic stroke, sympathetic nervous, catecholamine, neurogenic heart injury, cardiac injury

## Abstract

Acute ischemic stroke (AIS) may trigger a spectrum of cardiac complications spanning arrhythmias, troponin elevation, Takotsubo cardiomyopathy, heart failure, and myocardial fibrosis and other acute or chronic cardiac lesions. These complications seriously affect the prognosis of patients. Existing studies have shown that the excessive excitation of the sympathetic neural network after cerebral ischemic injury leads to an increase in catecholamine levels, which may be a key factor triggering neurogenic cardiac damage after AIS. Therefore, evaluating the trigger areas of sympathetic nerve excitation and monitoring related cardiac damage indicators play a key role in patient management. Inhibiting excessive excitation of the sympathetic nerve, alleviating inflammatory responses and oxidative stress, is expected to become the core strategy for the prevention and treatment of neurogenic cardiac injury after AIS. Future research still needs to deeply explore the mechanism of cardiotoxicity mediated by the sympathetic neuro-catecholamine system after AIS, and at the same time promote clinical trials targeting the mechanism to verify treatment paradigms through translational models. This review aims to provide a useful reference direction for subsequent in-depth research.

## Graphical Abstract

**Figure:**
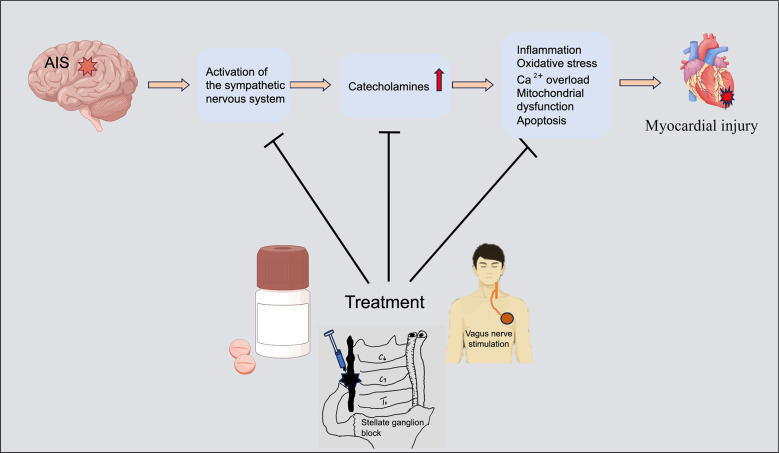
AIS activates the sympathetic nervous system (SNS), releasing a large amount of catecholamines, inducing myocardial inflammation, oxidative stress, Ca^2+^ overload, mitochondrial dysfunction and myocardial cell apoptosis, and ultimately causing myocardial damage. Curbing excessive excitement of SNS and reducing catecholamine toxicity can help alleviate myocardial injury. The figure was constructed with Figdraw (https://www.figdraw.com).

## 1 Introduction

Stroke is a neurological disease with high disability and mortality rates, and has risen to become the second leading cause of death worldwide (1). Acute ischemic stroke (AIS) is the most common type of stroke, and patients have often accompanied high risk for cardiac-related complications after the onset of stroke (2, 3). These cardiac complications are one of the common systemic complications after stroke and have second only to direct neurological impairment in terms of lethality (4, 5).Secondary cardiac injuries after AIS are manifested by acute or chronic cardiac arrhythmias, cardiac systolic dysfunction, elevated troponin (with or without myocardial ischemia), Takotsubo syndrome, sudden cardiac death, heart failure (HF), and myocardial fibrosis, among other acute or chronic cardiac pathologies (6–11). This cardiac damage has been shown to be associated with dysregulation of the autonomic nervous system (ANS) after stroke (4, 12–14), in particular hyperexcitability of the sympathetic nervous system (SNS) triggering catecholamine upregulation (15). Excessive catecholamines have adverse effects on the heart. For instance, norepinephrine (NE) and isoproterenol (ISO) have been observed to cause significant myocardial damage (16–18). This neurogenic cardiac damage may be due to the sustained activation of α1 adrenergic receptors (α1-AR) and β1 adrenergic receptors (β1-AR) by catecholamines, which leads to coronary artery constriction, accelerated heart rate, and hypertension, which in turn reduces myocardial perfusion (19, 20). Reduced myocardial perfusion triggers myocardial ischemia, which further induces elevated intracellular levels of calcium ions (Ca^2+^) and an increased level of reactive oxygen species (ROS), leading to myocardial injury and cardiomyocyte apoptosis (21). However, there is a lack of systematic summaries and updates on the mechanisms of cardiotoxicity caused by SNS overexcitation and catecholamine release after AIS. Therefore, further understanding of the potential mechanisms of cardiotoxicity by sympathetic nervous (SN) excitation as well as excess catecholamines is important for exploring the treatment of cardiac injury after AIS. In this review, we focus on the mechanisms of cardiotoxicity of catecholamine release triggered by SNS excitation after AIS, with the aim of providing new ideas and directions for clinical treatment.

## 2 Activation of the SNS after AIS

**Figure 1:**
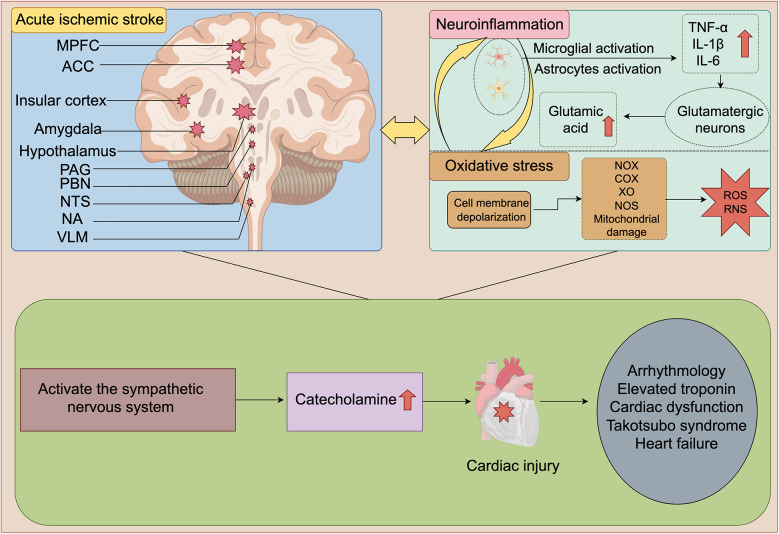
Schematic diagram of SNS activation and cardiac damage after AIS. Ischemic injury to MPFC, ACC, insular cortex, amygdala, hypothalamus, PAG, PBN, NTS, NA, and VLM is a key factor for SNS activation after AIS. After AIS, neuroinflammation and oxidative stress interweave with each other, jointly act and continuously activate the SNS, thereby leading to a sharp increase in catecholamine levels. Ultimately, it causes a series of cardiac damages such as arrhythmia, elevated troponin, cardiac dysfunction and HF. MPFC, medial prefrontal cortex; ACC, anterior cingulate cortex; PAG, periaqueductal gray; PBN, parabrachial nucleus; NTS, nucleus tractus solitarius; NA, nucleus ambiguus (NA); VLM, ventral lateral medulla; TNF-α, tumor necrosis factor-α; IL-1β, interleukin-1β; IL-6, interleukin-6; NOX, NADPH oxidase; COX, cyclooxygenase; XO, Xanthine oxidase; NOS, nitric oxide synthase; ROS, reactive oxygen species; RNS, Reactive Nitrogen Species. The figure was constructed with Figdraw (https://www.figdraw.com).

Some specific regions of the forebrain cortex, limbic lobe, and brainstem are collectively involved in central sympathetic nervous (CSN) regulation. However, the factors involved in SNS activation after stroke are complex and may involve mechanisms such as injury to key brain regions, inflammatory responses, and oxidative stress. Therefore, our study systematically summarizes the major factors and their potential mechanisms of SNS activation after AIS (Figure 1).

Figure 1 Schematic diagram of SNS activation and cardiac damage after AIS. Ischemic injury to MPFC, ACC, insular cortex, amygdala, hypothalamus, PAG, PBN, NTS, NA, and VLM is a key factor for SNS activation after AIS. After AIS, neuroinflammation and oxidative stress interweave with each other, jointly act and continuously activate the SNS, thereby leading to a sharp increase in catecholamine levels. Ultimately, it causes a series of cardiac damages such as arrhythmia, elevated troponin, cardiac dysfunction and HF. MPFC, medial prefrontal cortex; ACC, anterior cingulate cortex; PAG, periaqueductal gray; PBN, parabrachial nucleus; NTS, nucleus tractus solitarius; NA, nucleus ambiguus (NA); VLM, ventral lateral medulla; TNF-α, tumor necrosis factor-α; IL-1β, interleukin-1β; IL-6, interleukin-6; NOX, NADPH oxidase; COX, cyclooxygenase; XO, Xanthine oxidase; NOS, nitric oxide synthase; ROS, reactive oxygen species; RNS, Reactive Nitrogen Species.

### 2.1 Conduction pathways of the cardiac SNS

The SN is part of the ANS, which exhibits an extremely fine and complex anatomical configuration and functional layout in the brain and spinal cord. This intricate system, with the help of multiple neural network pathways, has had an important influence on the normal functioning as well as abnormal conditions of the cardiovascular system (14). The central autonomic nervous (CAN) network is structurally complex and includes projection pathways between the cerebral cortex, limbic system, and brainstem (22, 23). The SNS can be divided into the CSN network and the peripheral sympathetic nerve (PSN) network. While the CSN consists mainly of pathways that project from the paraventricular nucleus (PVN) of the hypothalamus to the rostral ventral lateral medulla (RVLM) and NE-containing cell populations in the pons (24–26). Among them, the RVLM, as a key center for the control of cardiovascular activity, plays a role in regulating heart rate and blood pressure by producing SN activity upon activation (27). These structures ultimately project to lateral horn motoneurons in the thoracic segment of the spinal cord and are transmitted via preganglionic fibers via the anterior spinal nerve roots and white traffic branches to the stellate ganglia of the parasympathetic trunk of the spinal cord, which subsequently emit postganglionic fibers that regulate cardiac activity (28, 29). In addition, neurons in the dorsal, ventral and lateral regions of the PVN and the RVLM can directly affect SN excitation by projecting directly to the medial-lateral aspect of the thoracic spinal cord (28, 30). Indeed, overexcitation of the SN has been a major concern for cardiac injury after AIS, and a large number of studies have revealed that overactivation of the CSN triggers cardiac injury after stroke (31–34).

**Figure 2:**
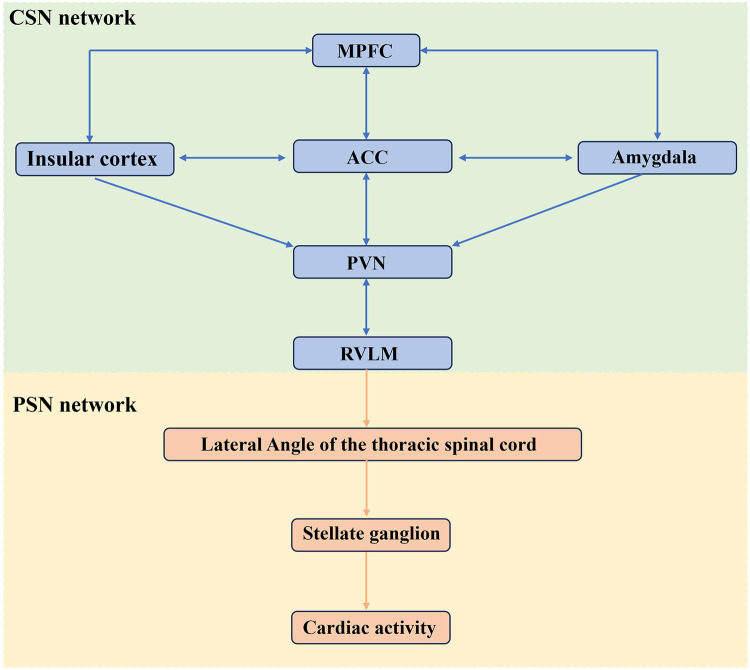
The **SNS** governing cardiac activity. Cardiac activity is jointly regulated by CSN and PSN, with the RVLM serving as a critical hub for cardiovascular control. MPFC, medial prefrontal cortex; ACC, anterior cingulate cortex; RVLM, rostral ventral lateral medulla; PVN, paraventricular nucleus. The figure was constructed with Microsoft PowerPoint.

Earlier, researchers recognized that the hypothalamus, insula cortex had the function of regulating SN activity (24, 35). With the development of neuroanatomy as well as imaging techniques, the ventral medial prefrontal cortex (vMPFC), insula, anterior cingulate cortex (ACC), hypothalamus, amygdala, and brainstem were found to be involved in the regulation of the ANS (22, 29, 36). Under stress, hypoxia, hypovolemia, and hypoglycemia, the vMPFC, insula, and ACC could activate the SNS by activating RVLM glutamatergic neurons via downward conductance (29). In addition, RVLM glutamatergic neurons can also be directly activated by these factors to trigger SN excitation (29). However, there was no comprehensive and systematic summary study on how SNS to be activated after AIS. Therefore, the present study aims to systematically summarize the activation factors of the CSN after AIS, with the aim of providing a clear framework for an in-depth understanding of the activation mechanisms of the CSN network after stroke. Figure 2 illustrates the SNS that regulates cardiac activity.

Figure 2 The **SNS** governing cardiac activity. Cardiac activity is jointly regulated by CSN and PSN, with the RVLM serving as a critical hub for cardiovascular control. MPFC, medial prefrontal cortex; ACC, anterior cingulate cortex; RVLM, rostral ventral lateral medulla; PVN, paraventricular nucleus.

### 2.2 Impairment of the CAN network conductance pathway triggers increased SN tension

Ischemia in some key regions is more likely to trigger cardiac damage, which is closely related to brain regions associated with the ANS projection loop. Specifically, ischemic cerebral infarction may activate the SNS in direct or indirect ways. For example, elevated SN tone in response to damage to key structures involved in parasympathetic (PN) projections may lead to hyperactivation of the SNS. This phenomenon suggests that lesions in specific regions of the brain have an important impact on the balance of the ANS.

#### 2.2.1 Medial prefrontal cortex (MPFC)

Clinical researches have shown that frontal lobe strokes often cause arrhythmias like atrial fibrillation (AF), tachycardia, and ventricular arrhythmias (37, 38). Using functional magnetic resonance imaging to explore the relationship between heart rate and cortical activity found that increased heart rate or increased heart rate variability in healthy volunteers is associated with diminished MPFC activity (36, 39). Transcranial direct current stimulation of the MPFC induced post-exercise hypotension in subjects (40). These conditions suggest that the MPFC is involved in the regulation of the ANS (41). Animal experiments indicate that the vMPFC regulate acute restraint—induced tachycardia in rats. The infralimbic cortex promoted CoCl_2_-induced tachycardia, while the prelimbic cortex have the opposite effect (42). Additionally, electrically stimulating the rat MPFC significantly increased blood pressure (43). In humans, studies on patients with vMPFC lesions in different locations have showed that the left vMPFC is linked to PN activation, and the right vMPFC to SN inhibition (44). This lateralized regulation of the ANS may clarify why right MPFC damage activates the SN.

#### 2.2.2 Insular cortex

The insular is often regarded as a key area for AIS—induced cardiac damage. Many clinical studies indicated that insular cortex involvement in ischemia often results in cardiac problems liked bundle branch block, arrhythmias, QT-interval prolongation, myocardial injury, and cardiac dysfunction (45–50). A study of 384 infarcts in the middle cerebral artery region found that patients with insula damage showed elevated levels of NE and neutrophils (51). Further comparison of right and left insula damage revealed that patients with right insula damage exhibited reduced heart rate variability (51, 52). Also, right insular damage and arrhythmia due to SN activation became an adverse factor affecting 1-year prognosis (46). Many studies have shown right insular damage was more likely to cause cardiac lesions, especially arrhythmias (13, 53–58), while left damage would lead to elevated troponin and BNP (59). These results implied right insular damage activated the SNS. A plausible explanation was that after right insula infarction, reduced PN tension and pressure reflex sensitivity allowed the SN to dominate autonomic nervous(AN) regulation (60). In a study by Oppenheimer SM and colleagues on patients undergoing epilepsy surgery, it had found that stimulation of the left insular cortex resulted in a slowing of the heart rate, while stimulation of the right insular cortex had the opposite effect (61). This indicated that the left insular was associated with the regulation of the PN system, and the right insular with the SNS. Animal of middle cerebral artery occlusion (MCAO) models also have confirmed that right insular damage led to increased plasma NE and QT-interval prolongation (62, 63). These experimental results support the existence of a lateralized effect of insula damage on cardiac regulation, which may be due to the dominant role of the right insula in controlling the PN downstream conduction pathway to the sinus node (64). Moreover, the insular cannot directly project to sympathetic preganglionic neurons; its regulation of the SNS likely requires integration through hypothalamic nuclei. For example, the glutamatergic relay in the dorsomedial hypothalamus (DMH) has been proven to be a pathway for its regulation of the SNS (34). Some studies have shown that the DMH exhibits asymmetry in the regulation of cardiac AN function, with the right DMH primarily responsible for regulating cardiac rhythm (65, 66). When the right insular is damaged, it may trigger dysregulation of the SNS via the DMH pathway, leading to arrhythmias. However, research on the mechanisms linking insular lesion location and hemispheric laterality to AN dysregulation and cardiac damage remains very limited. Further in-depth studies will help to better understand this area.

#### 2.2.3 Limbic system and brainstem

Limbic systems such as ACC, amygdala and hypothalamus can modulate cardiac activity through direct or indirect or periaqueductal gray (PAG) relay projections to the medulla oblongata and lateral horn of the spinal cord (29). In addition, the nucleus tractus solitarius (NTS), ventral lateral medulla (VLM), nucleus ambiguus (NA) and parabrachial nucleus (PBN) in the brainstem are also involved in their network connections (60). The ACC is interconnected with the insula cortex. The ACC mainly modulates the SN and PN systems via its ventral and dorsal regions (67, 68), with the left side predominantly engaging in parasympathetic regulation (69).

During stress, excess NE heightens amygdala—induced excitatory regulation of the SNS, strengthening SN activity (70). The amygdala, with its extensive neural connections to the hypothalamus and brainstem, modulates sympathetic preganglionic neuron activity via these pathways, impacting sympathetic output. Thus, the amygdala is crucial for regulating SNS and neuroendocrine responses to stress (71, 72). The hypothalamus plays a central role in maintaining the stability of the body's internal environment by regulating the ANS, and its anterior and posterior regions are representative of the PN and SN (73). And the paraventricular nucleus (PVN) of the hypothalamus can project directly or indirectly to the preganglionic neurons of the SN and play a role in the regulation of cardiac activity by afferent sensory signals from the heart via the NTS (74). The PAG, NTS, VLM, NA and PBN in the brainstem are key structures and relay stations in the ANS that connects the forebrain, limbic system and spinal cord (60, 75–77). These structures are involved in cardiovascular regulation, and their damage can cause severe SN activation (78–80). Numerous studies have shown that lesions to these structures lead to activation of the SNS, which can lead to a range of cardiac problems, especially arrhythmias (77, 81–88).

In summary, ischemic damage to central regions regulating the SN and PN networks may be excessive activation of SN, disrupting cardiac physiology and causing damage. Such patients should be closely monitored in clinical settings. Although a large number of studies have reported the relationship between the lateralization of brain injury sites and cardiac damage, how this lateralization regulates the activation of the SNS still lacks in-depth discussion and needs to be explored and elucidated by more studies.

### 2.3 Neuroinflammation and oxidative stress promote the activation of SN after AIS

A large number of studies have observed that neuroinflammation and oxidative stress are important factors causing or aggravating brain tissue injury after stroke (89–91). For example, pro-inflammatory factors (e.g., tumor necrosis factor-α (TNF-α), interleukin-1β (IL-1β), and interleukin-6 (IL-6), etc.) (92–95), inflammatory mediators (e.g., prostaglandins (PGE2), nuclear kappa factor B (NF-κB), and cyclooxygenase-2 (COX-2), etc.) (96, 97), and oxidative stress-associated molecules (e.g., ROS, RNS, and MDA, etc.) are upregulated after stroke (98, 99).

Inhibiting these factors and oxidative stress levels can significantly improve brain injury. After AIS occurs, central inflammatory cells such as astrocytes and microglia will be rapidly activated, releasing a large amount of inflammatory factors (such as TNF-α, IL-1β and IL-6) and matrix metalloproteinases, causing damage to the blood-brain barrier (89). Meanwhile, these cells promote the infiltration of peripheral immune cells (such as neutrophils, macrophages and lymphocytes) into the ischemic area by up-regulating the expression of cell adhesion molecules (such as ICAM-1 and selectin), thereby exacerbating neuroinflammation and oxidative stress injury (91). When blood flow is interrupted, the brain can't get energy from glucose oxidative phosphorylation and instead uses fatty acids. This leads to lipid peroxidation, producing lots of ROS and reactive nitrogen species (RNS). They damage cell membranes and cause neuronal injury (100). Oxidative stress not only directly leads to cell damage, but also activates inflammatory responses. For example, ROS and RNS can activate transcription factors (such as NF-κB), thereby promoting the production of pro-inflammatory factors and further aggravating neuroinflammation (89). The resulting brain injury may be an important factor for the activation of the SNS (101, 102).

Following AIS, the release of pro—inflammatory cytokines activates the SNS, causing the adrenal medulla and sympathetic nerve terminals to release catecholamines (103). This can lead to coronary artery constriction and myocardial ischemia, as well as activate peripheral monocytes/macrophages and neutrophils, thereby exacerbating cardiac injury (6). Inhibiting microglial cell-mediated neuroinflammation can improve ventricular arrhythmias in HF rats (104). In a canine MCAO model, ventricular tachycardia (VT) occurrence is linked to heightened left stellate ganglion activity, elevated NE levels, and activated M1-type microglia in the ventricle, along with increased TNF-α, NF-κB, and MCP-1 levels. These phenomena can be significantly diminished by ablating the left stellate ganglion (105). This may be related to the increase in SN activity caused by pro-inflammatory factors such as IL1-β, TNF-α, and IL-6 stimulating glutaminergic neurons to secrete glutamic acid (106, 107). In addition, orexin A (OXA) in rat PVN can promote the expression of IL1-β, IL-6, and TNF-α through orexin 1 receptor (OX1R) and increase SN activity (108).

However, few studies have explored the relationship between oxidative stress and SNS activation after AIS. Several studies have illustrated from the side the connection between central oxidative stress and the SNS as well as cardiac injury. Inducing the overexpression of inducible nitric oxide synthase (iNOS) in RVLM caused SN excitation in rats and increased NE production (109). The use of long-acting calcium dihydropyridine channel blockers can inhibit SN activity and the oxidative stress level of RVLM and increase the ability to resist oxidative stress (110). When atropine is administered to inhibit PN system activity, MCAO mice exhibit more severe cardiac injury, characterized by reduced left ventricular ejection fraction, increased myocardial apoptosis, and fibrosis. Concurrently, the expression of the antioxidant factor endothelial nitric oxide synthase (eNOS) is decreased (9). Renal denervation can reduce the release of catecholamines and inhibit the expression of NADPH oxidase in the brain of rat models at high risk of stroke (111). These findings indicate that neuroinflammation and oxidative stress enhance SN excitation after AIS and induce cardiac damage.

## 3 Cardiotoxicity triggered by catecholamines

After stroke, increased SN tension elevates circulating catecholamine levels, subsequently inducing cardiac dysfunction (112). These catecholamines primarily consist of epinephrine (E) and NE (11). NE is primarily synthesized by noradrenergic neurons in the central nervous system and postganglionic sympathetic neurons (113, 114), which serve as neurotransmitters in the regulation of intracranial signals and the control of peripheral target organs such as the heart and vasculature. In the adrenal medulla, tyrosine undergoes a series of enzymatic reactions to gradually convert into NE. Ultimately, under the catalysis of phenylethanolamine N-methyltransferase, a significant portion of NE is converted into E (115), which is released into the bloodstream in a hormonal form to participate in the regulation of physiological functions. However, the plasma NE primarily originates from the release of neurotransmitters from the terminals of postganglionic sympathetic neurons, not from the adrenal medulla. Therefore, NE functions both as a classical neurotransmitter in the synaptic cleft and as a hormone via the bloodstream, reflecting its dual role in physiological regulation.

Many clinical investigations and animal studies have demonstrated catecholamine surges after stroke (6, 77, 81, 116). Excess catecholamines can induce coronary artery constriction, raise heart rate, and increase myocardial oxygen consumption. This can lead to myocardial inflammation, oxidative stress, Ca^2+^ overload, mitochondrial dysfunction, and myocardial cell apoptosis. In conclusion, these processes are very complex. Our study has summarized the cardiotoxicity of catecholamines and its mechanism (Figure 2).

**Figure 3:**
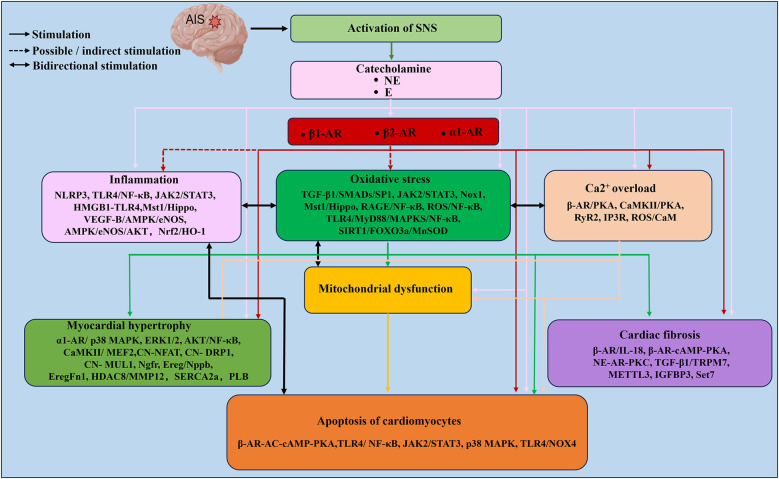
The mechanism of catecholamine-triggered cardiotoxicity after AIS. After AIS, sympathetic nerve tension increases, and sustained overexcitation of the sympathetic nerve network leads to substantial catecholamine release. The activation of AR, inflammatory response, oxidative stress, Ca^2+^ overload and mitochondrial dysfunction constitute the main network of catecholamine-induced cardiac injury. These pathological processes are interrelated. Centered on oxidative stress, they form a complex network through multiple signaling pathways, eventually leading to cardiomyocyte apoptosis, myocardial hypertrophy and cardiac fibrosis. The relevant signal pathways or key molecules are marked below each topic in the text box. The figure was constructed with Microsoft PowerPoint.

Figure 3 The mechanism of catecholamine-triggered cardiotoxicity after AIS. After AIS, sympathetic nerve tension increases, and sustained overexcitation of the sympathetic nerve network leads to substantial catecholamine release. The activation of AR, inflammatory response, oxidative stress, Ca^2+^ overload and mitochondrial dysfunction constitute the main network of catecholamine-induced cardiac injury. These pathological processes are interrelated. Centered on oxidative stress, they form a complex network through multiple signaling pathways, eventually leading to cardiomyocyte apoptosis, myocardial hypertrophy and cardiac fibrosis. The relevant signal pathways or key molecules are marked below each topic in the text box.

### 3.1 The molecular mechanisms of catecholamine-mediated cardiac injury

#### 3.1.1 Cardiac inflammation and oxidative stress induced by catecholamines

In Takotsubo syndrome, AIS, and ISO-induced myocardial infarction animal models, a significant inflammatory response was observed in the heart, as evidenced by infiltration of inflammatory cells and upregulation of inflammatory factor levels (117–120). A large number of clinical, animal and cellular experiments demonstrated that post-stroke cardiac injury was closely related to inflammation, and that the main mechanism involved the up-regulation of pro-inflammatory factors and mediators, as well as the infiltration of inflammatory cells such as monocytes, macrophages, and lymphocytes (6, 19, 121–123). Researchers have long noted that stress-generated catecholamines may lead to cardiac damage and observed significant myocardial inflammation (124), and a positive correlation between leukocyte levels and NE levels (125). With the development of magnetic resonance imaging (MRI) techniques, significant macrophage infiltration of the myocardium has been observed in patients with Takotsubo syndrome (121). Animal experiments often use a model of ISO-induced cardiac damage. ISO is a β-AR agonist and has a chemical structure similar to that of biogenic amines and is often used as a synthetic model to study catecholamine toxicity. Several studies have found that elevated catecholamine levels, left ventricular inflammatory infiltration and myocardial fibrosis were observed in both ISO-induced and chronic stress-induced rat Takotsubo models (126). These results suggest that elevated catecholamines after AIS may be an important factor contributing to cardiac inflammation.

Several studies have reported several mechanisms to mimic catecholamine-induced cardiac inflammation with ISO: mainly Nod-like receptor protein 3 (NLRP3), TLR4/NF-κB pathway, JAK2/STAT3 pathway, and HMGB1-TLR4 signaling. SN excitability activates the NLRP3 vesicles in the heart and generates IL-1β to induce myocardial injury (127), and recent studies have also demonstrated that cardiac insufficiency occurring after MCAO in mice is associated with sustained pro-inflammatory changes in monocytes/macrophages driven by IL-1β (122). In the ISO-induced male mouse model of stress cardiomyopathy, ISO triggers NLRP3 inflammasome activation via NOX4-dependent mitochondrial ROS generation, up-regulates downstream inflammatory cytokines including IL-6 and TNF-α, and promotes recruitment of CD68^+^ CD11b^+^ macrophages into the myocardium (128). ISO also stimulates NLRP3 inflammasome via NOX4-dependent mitochondrial ROS and activates downstream inflammatory signals (e.g., IL6 and TNFα) while inducing infiltration of CD68 and CD11b-expressing macrophages into the myocardium of mice (129). In addition, MD2 is also activated by β1-AR-ROS signaling and induces macrophage polarization to generate inflammation via the β2-AR-cAMP-PKA-ROS axis (130). HK1 has also been found to activate NLRP3 in the myocardium of ISO-treated mice (131). This suggests that HK1, MD2 may also be a pathway that induces cardiac inflammation.

TLR4 has been shown to be extensively involved in stroke, myocardial infarction and inflammation, where its binding to the bridging molecules MyD88 or MAPK activates NF-κB and triggers the up-regulation of IL-1β, IL-18 and TNF-α expression (132–134). On the one hand, ISO can upregulate Gal-3 expression and induce myocardial inflammation and fibrosis via the TLR4/ MyD88/NF-κB pathway, and the use of the Gal-3 blocker MCP ameliorates this adverse outcome (135). On the other hand, myocardial inflammation and apoptosis can also be induced through the AMPK/NF-κB pathway (136).

The JAK2/STAT3 signaling pathway is also involved in ISO-induced myocardial inflammation and hypertrophy. Upon activation, it drives macrophages to polarize into a pro-inflammatory phenotype and induces the release of pro-inflammatory factors, and JAK2 inhibitors ameliorate myocardial inflammation (137). High mobility group protein 1 (HMGB1) is also found to be upregulated in expression in ISO-treated rat hearts and trigger inflammation through the HMGB1-TLR4 pathway (138). Several other signaling pathways, such as Mst1/Hippo, VEGF-B/AMPK/eNOS, AMPK/eNOS/AKT, and Nrf2/HO-1, have recently been found to be involved in the regulation of cardiac inflammation after ISO treatment (139–142).

The metabolism of NE has an important role in oxidative stress, such as ROS production (143). NE is eliminated mainly through presynaptic and extraneuronal reuptake as well as metabolism. First, oxidative deamination by monoamine oxidase (MAO) converts NE to dihydroxyphenylethanol. Then, it is converted to methoxyhydroxyphenylglycol (MHPG) catalyzed by catechol-O-methyltransferase (COMT). It is finally converted to 3-methoxy-4-hydroxymandelic acid (VMA) and 3-methoxynorepinephrine (NMN) in the liver and excreted via urine (144–146). Catecholamine-induced oxidative stress injury has been demonstrated in the heart (147). It can lead to increased lipid peroxidation, and several antioxidants can attenuate the damage caused by lipid peroxidation (148–150). For example, after ISO injection, the levels of antioxidant enzymes SOD and glutathione peroxidase (GSH-Px) in the myocardium of rats decreased significantly, while the contents of oxidative stress markers MDA and NO increased significantly. Inhibiting oxidative stress can effectively alleviate heart damage (120, 142). Cardiomyocytes treated with E produced hydroxyl radicals (⋅OH) and peroxynitrite (ONOO^−^) (151). The expression of antioxidants such as SOD, GSH-Px and catalase was also found to be decreased in H9c2 cardiomyocytes exposed to NE, while the expression of 4-hydroxynonenal was upregulated (152). These results suggest that catecholamine-induced oxidative stress is also an important cause of cardiac injury. In addition, when liver function is impaired, the activities of MAO and COMT decrease, and the ability to generate and clear catecholamine metabolites (such as VMA) declines, which may lead to the accumulation of these metabolites in the body (153–155). Similarly, when the kidneys are damaged, especially when the glomerular filtration rate (GFR) decreases, it can also lead to a reduction in the excretion of catecholamine metabolites, further intensifying the accumulation of these metabolites in the body (156). Therefore, when renal or hepatic dysfunction is present, the impaired clearance of these metabolites may act as a “second hit” to cardiac injury. However, current research on the association between the toxic byproducts of catecholamine metabolism and cardiac injury after stroke remains insufficient. Clarifying these relationships may be of significant importance for elucidating the mechanisms underlying neurogenic cardiac injury after stroke.

The conditions that trigger oxidative stress include multiple complex factors such as mitochondrial damage, inflammation, apoptosis and oxidative damage to proteins, lipids and DNA (11). Among these factors, ROS production is the main driver of oxidative stress. The source of catecholamine-induced ROS is multifactorial in nature and consists of the following two main aspects: (1) Stimulation of the AR: for example, in cardiac myocytes NADPH oxidase is activated by α1-AR stimulation, which leads to the generation of superoxide anion radicals (O_2_−•) (157). (2) Enzymatic and non-enzymatic degradation of catecholamines: the MAO pathway induces oxidative deamination of NE to produce hydrogen peroxide (H_2_O_2_) which is further catalyzed to “⋅OH”; and degradation of NE by non-enzymatic pathways produces “aminochromes” toxic compounds (11, 158). This catecholamine-mediated oxidative stress causes aberrant cell signaling, intracellular Ca^2+^ overload, mitochondrial damage, inflammatory responses, and disruption of the extracellular matrix and lysosomes, which in turn triggers apoptosis in cardiomyocytes (159–163). Also, these adverse outcomes directly or indirectly enhance oxidative stress, ultimately causing arrhythmias, cardiac hypertrophy, myocardial fibrosis, cardiac insufficiency, and HF. These results suggest that catecholamine-induced oxidative stress may be central to cardiac injury.

Recent study indicates that renal denervation decreases catecholamine secretion in hypertensive HF rat models, lowering ROS and MDA levels and reducing myocardial hypertrophy and fibrosis. This may be related to the inhibition of BACH1 by the TGF-β1/SMADs/SP1 signaling pathway and the alleviation of mitochondrial oxidative stress by PACS-2 (164). However, there is currently a lack of more research to explain how renal denervation directly inhibits specific molecular pathways within the heart. This may be related to the fact that renal denervation reduces sympathetic nerve activity and catecholamine levels throughout the body, and it is precisely these systemic changes that ultimately affect the signal transduction pathways of the heart. In both ISO—treated mice and cardiomyocytes, p-JAK2, p-STAT3, MDA, NOX2, and NOX4 show increased expression, yet inhibitors can reverse this trend (137). Thus, JAK2/STAT3 signaling plays a dual role in ISO-induced oxidative stress and inflammation. In addition, the Mst1/Hippo, RAGE/NF-κB, ROS/NF-κB, TLR4/MyD88/MAPKS/NF-κB, and SIRT1/FOXO3a/MnSOD signaling pathways are also found to be involved in ISO-induced oxidative stress injury in the heart (139, 165–168). Activation of these signaling pathways may in part explain the mechanisms of catecholamine-induced cardiac inflammation and oxidative stress. However, these processes remain complex and can influence each other, and more mechanistic studies are needed.

#### 3.1.2 Ca^2+^ overload in cardiomyocytes

Ca^2+^ is a key regulatory ion in cardiac excitation-contraction coupling. Excessive release of catecholamines sustains activation of the β-AR, leading to a significant increase in myocardial excitability and contractility. β-AR overactivation may trigger intracellular Ca^2+^ overload, which leads to a series of cardiomyocyte injuries. Meanwhile, Ca^2+^ overload activates Ca^2+^-dependent ATPase, leading to mitochondrial dysfunction and increased oxidative stress, which in turn triggers cardiomyocyte injury (169).

A significant increase in the level of Ca^2+^ in myocardial cells is observed in the ISO-induced myocardial ischemia model (170). Further studies have shown that ISO promotes Ca^2+^ transients and increases Ca^2+^ load in the myocardial sarcoplasmic reticulum (SR) via β-AR (171). In addition, ISO can cause Ca^2+^ overload via L-type calcium channels (LCC) (172). In cardiomyocytes, NE activates β1-AR, which in turn promotes Ca^2+^ endocytosis via LCC and triggers Ca^2+^ release from the SR via the ryanodine receptors (RyR2) pathway (173). Continuous accumulation of Ca^2+^ may continuously activate calcium ion-dependent ATPase, thereby damaging mitochondrial oxidative phosphorylation function. This impairment would further exacerbate the disturbance of intracellular Ca^2+^ homeostasis, disrupt normal energy metabolism, promote ROS accumulation, and ultimately lead to myocardial excitation-contraction dysfunction (169, 174). Intracellular Ca^2+^ overload is closely associated with the development of arrhythmias and may also directly induce cardiomyocyte apoptosis (175). This may be due to the opening of the mitochondrial permeability transition pore after impaired mitochondrial function, which triggers apoptosis (176).

Furthermore, oxidative stress can also cause impaired mitochondrial function, resulting in insufficient ATP production, ATP-dependent Na^+^-Ca^2+^ exchange disorders, and promoting Ca^2+^ accumulation (177). When 5-AR is activated, calmodulin-dependent protein kinase II (CaMKII) regulates calcium channels and RyR2 via PKA-dependent phosphorylation, thereby driving Ca^2+^ accumulation (146). Recently study found that 4-hydroxyketone, produced by NE metabolized by mitochondrial MAO-A, promotes Ca^2+^ accumulation through the voltage—dependent anion channel 1/inositol-1,4,5-trisphosphate receptor 1 (IP3R) pathway (178). ROS generated by NE metabolism cause intracellular Ca^2+^ overload either by modulating calcium—handling proteins or inducing membrane lipid peroxidation (143). The resulting mitochondrial Ca^2+^ accumulation disrupts the mitochondrial membrane potential and damages the respiratory chain, further boosting ROS production (11). This vicious cycle may exacerbate cardiomyocyte injury.

#### 3.1.3 Mitochondrial dysfunction

In numerous animal experiments, catecholamine-induced cardiomyocyte mitochondrial dysfunction has been observed. The ROS generated during catecholamine metabolism, such as O^2^−• and H_2_O_2_, can directly attack the lipids, proteins, and DNA of mitochondria. Consequently, the structure and function of mitochondria become impaired, and their normal oxidative phosphorylation process is affected (179, 180). Another product of the metabolic process, dopaldehyde and 3,4-dihydroxyphenylacetaldehyde, can also interfere with the normal physiological function of mitochondria, or even destroy the structure of mitochondria, leading to mitochondrial dysfunction (179). Moreover, catecholamine-induced mitochondrial Ca^2+^ overload inhibits mitochondrial respiration and ATP synthesis, and increases mitochondrial permeability (11). Ca^2+^ overload can cause mitochondrial dynamism abnormalities, which may be related to the acetylation of ATPase family AAA domain—containing protein 3A (181). ISO can reduce the expression of antioxidant enzymes such as SOD and CAT in mitochondria, thereby increasing mitochondrial oxidative stress levels (182).

Cardiomyocyte mitochondrial swelling and myofilament vacuolization were observed in rats after ISO treatment (183). Further analysis revealed that the respiratory control index reflecting oxidative phosphorylation (184), the cardiac phosphocreatine/ATP ratio, and ATP content were all reduced, while MDA and eNOS expression increased (185, 186). This indicates that ISO induced energy production impairment in the myocardium. Some studies have shown that after ISO intervention, mitochondrial dysfunction in the heart is associated with reduced expression of certain enzymes, including mitochondrial respiratory enzymes [such as NADH dehydrogenase, succinate dehydrogenase (SDH), and cytochrome c oxidase (CcO)] (187), aldehyde dehydrogenase 2 (ALDH2), and the mitochondrial enzyme β-hydroxyacyl-CoA dehydrogenase (HADH) (188).

Mitochondrial respiratory enzymes are crucial for normal mitochondrial physiological functions (189). NADH dehydrogenase, the first enzyme complex in the mitochondrial electron transport chain, mainly transfers electrons from NADH to coenzyme Q (CoQ) while pumping protons from the mitochondrial matrix into the intermembrane space to form a proton gradient for ATP synthesis. SDH, complex II of the respiratory chain, passes electrons from FADH2 to CoQ, linking the tricarboxylic acid cycle to the electron transport chain. As the terminal oxidase in the chain, CcO receives electrons from cytochrome C(Cyt-C) and transfers them to oxygen, completing the final step of electron transport and driving ATP synthesis. The reduction of NADH, SDH and CcO activities jointly weakens the oxidative phosphorylation efficiency of mitochondria, reduces ATP production, thereby leading to insufficient energy supply to cardiomyocytes and further affecting the contractile and diastolic functions of the myocardium (190). In addition, energy metabolism disorders can cause mitochondrial dysfunction, leading to an increase in ROS production and subsequently damaging the mitochondrial membrane and DNA. This kind of damage will further disrupt mitochondrial function and promote the release of Cyt-C, activate the caspase cascade reaction, and ultimately induce apoptosis of cardiomyocytes (190, 191).

ALDH2 is involved in metabolizing reactive aldehydes produced during oxidative stress and exerts cardioprotective effects by inhibiting oxidative stress and inflammationALDH2 (192). ALDH2-knockout mice show aggravated cardiac ischemia-reperfusion injury (193). HADH, which participates in fatty acid β-oxidation, causes abnormal energy production when reduced, as it impedes fatty acid oxidation (194). The study has found that SFRP4 is involved in ISO-induced cardiomyocyte mitochondrial damage, and using an SFRP4 inhibitor can alleviate mitochondrial dysfunction in the myocardium and HL-1 cells (195). In summary, cardiomyocytes have a high energy demand, and mitochondrial dysfunction may play a significant role in the development of neurogenic cardiomyopathy after AIS. Impaired mitochondrial function leads to disordered energy metabolism and reduced ATP production in cardiomyocytes. This exacerbates the imbalance of calcium ion homeostasis, ultimately causing cardiomyocyte apoptosis and necrosis and worsening myocardial injury (182, 183).

#### 3.1.4 Apoptosis of cardiomyocytes

At physiological concentrations, the toxicity of catecholamines to cardiomyocytes is generally insignificant. However, excessive catecholamines may induce cardiomyocyte apoptosis. It should be noted that the longer the exposure time of cardiomyocytes to catecholamines, the more pronounced the potential damaging effects may become. In a large number of animal and cell experiments, it has been observed that NE and ISO can induce cardiomyocyte apoptosis (16, 152, 196). They achieve this by modulating the expression of Bcl-2 family proteins, for instance, enhancing Bax expression and suppressing Bcl-2 and Bcl-XL expression (197). In addition, NE and ISO can upregulate Cyt-C expression. By activating caspases (including caspase-2, caspase-3, caspase-6, and caspase-9) and death receptors [such as Fas and TNF receptor 1-associated death domain (TRADD)], and enhancing the activity of apoptotic protease activating factor-1 (Apaf-1), NE and ISO mediate cardiomyocyte apoptosis (197–200).

The Bcl-2 family plays a crucial regulatory role in apoptosis, involved in both mitochondrial and some extrinsic apoptotic pathways. It consists of two main subtypes: anti-apoptotic proteins like Bcl-2, Bcl-XL, and Bcl-w, and pro-apoptotic proteins, which include BAK, BAX, BOK, and BH3-only proteins (201). In rat H9C2 cardiomyocytes treated with NE, Hoechst fluorescence staining showed increased apoptosis, with upregulated BAX and downregulated Bcl-2 expression (202). n ISO-induced HF models, rats exhibited increased Bax, Cyt-C, Caspase-3, and Caspase-9 expression, alongside decreased Bcl-2 and Bcl-XL expression in the myocardium (203). BAX forms a heterodimer with Bcl-2, reducing its activity. This increases mitochondrial membrane permeability, releasing Cyt-C into the cytosol. Cyt-C binds to Apaf-1 to form an apoptosome, triggering the Caspase cascade, leading to cell destruction and apoptosis (201, 204). Conversely, Bcl-2 protects cells by inhibiting Cyt-C release (205). Overall, NE and ISO induce cardiomyocyte apoptosis via the mitochondrial apoptosis pathway mediated by the Bcl-2 family.

Following ISO treatment, rats exhibited remarkable myocardial injury, with upregulated cardiac expression of Fas and caspase-3/8/9, and TNF-α genes (120, 206, 207). Mice with TNF receptor 1 (TNFR1) knockout showed resistance to ISO-induced cardiac injury, marked by downregulated expression of IL-1 β, iNOS, NF-κB, and AP-1 (208). This implies the death receptor family participates in ISO-mediated cardiomyocyte apoptosis. After TNF α binds to TNFR1, its intracellular death domain recruits TRADD. This facilitates the assembly of signaling molecules like receptor-interacting protein kinase 1, Fas-associated death domain protein, and caspase-8, forming complex I. This process activates downstream genes (e.g., the caspase cascade) and promotes complex II formation, inducing apoptosis (199).

ISO also activates the TLR4/NF-κB and JAK2/STAT3 signaling pathways, triggering inflammatory responses that interact with apoptosis (135, 137). Additionally, other upstream signals participate in ISO-induced cardiomyocyte apoptosis. For example, TLR4/NOX4, p38 MAPK, and Jak1/Stat signaling are activated (209–211), affecting Cyt-C release, activating caspases, and mediating apoptosis via the mitochondrial apoptosis pathway. The β-AR-AC-cAMP-PKA pathway can activate transcription factors like CREB, promoting pro-apoptotic gene expression, and also induces cardiomyocyte apoptosis through the mitochondrial apoptosis pathway (212). ER stress and CaMKII-mPTP can cause Ca^2+^ overload and promote apoptosis in the same way (213, 214).

### 3.2 Catecholamine-induced structural cardiac injury

#### 3.2.1 Myocardial hypertrophy

Many studies have confirmed that sustained activation of the SNS is closely related to cardiac hypertrophy (CH) (18, 215, 216), and that renal denervation of the SN can improve CH (164). This may be closely related to the increased secretion of catecholamines caused by SN hyperactivity (202, 217). Continuous pressure load can induce CH. This is an adaptive manifestation, but long-term pressure loads can lead to the generation of HF. CH is characterized by an increase in the heart weight—to—body weight ratio (HW/BW). Hematoxylin-eosin staining shows an increase in the cross-sectional area of cardiomyocytes, often accompanied by increased expression of CH-related genes such as BNP, β-MHC, and ANP (218). In animal HF models induced by NE and ISO, significant CH has been observed (219, 220). This may be related to the activation of signaling pathways such as MAPK, NF-κ B, Ca2+, JAK2/STAT3, G protein-coupled receptor kinases (GRKs), and Protein Kinase A (PKA).

When NE binds to cardiac α1-ARs, it activates p38 MAPK and ERK1/2, upregulating genes related to CH. NF-κB signaling activation induces myocardial inflammation and promotes CH (221). In a mouse CH model induced by NE, significant increases in the phosphorylation levels of p38, MAPK, ERK1/2, AKT, and NF-κB proteins occur in myocardial tissue, with notable upregulation of CH-related genes like ANP, BNP, and β-MHC. Moreover, pharmacological inhibition of the p38 MAPK/ERK1/2 and AKT/NF-κB pathways significantly attenuates CH in mice (218).

Ca^2+^ regulates CH through the calcineurin-NFAT and CaMKII-MEF2 pathways. When NFAT in the cell membrane is activated by calcineurin and translocated to the nucleus. It then interacts with nuclear transcription factors like GATA-4 and MEF2, upregulating the transcription of CH-related genes (222, 223). Interestingly, Ca^2+^ can activate the CaMKII*δ*B/CREB pathway, increasing mitochondrial Ca2+ uniporter(MCU) expression to alleviate CH (224). But recent studies show that MCU3 overexpression promotes Ca^2+^ uptake and induces CH (225). Thus, MCU upregulation may be a compensatory mechanism where different MCU subunits interact to regulate Ca^2+^ homeostasis. Calcineurin can also activate the pathway involving dynamic-related protein-1, upregulating mitochondrial E3 ubiquitin ligase 1 expression. This promotes mitochondrial fission and dysfunction, leading to CH (226, 227).

Some studies have indicated that JAK2/STAT3 signaling activation upregulates genes tied to CH. Activated STAT3 increases expression of ANP, BNP, and β-MHC, triggering CH. It also modulates AMPKα/mTOR signaling, influencing cardiomyocyte metabolism and autophagy, and thus CH regulation (202, 228). GRKs mainly impact cardiomyocyte signal transduction by regulating G protein-coupled receptor activity. Key GRKs like GRK2 and GRK5 play important roles. GRK2 influences cardiomyocyte survival and hypertrophy by regulating PI3K/AKT signaling (229). GRK5 enhances NFAT transcriptional activity, upregulating CH-related gene expression (230). SN-induced catecholamine release, binding to β-AR, activates adenylate cyclase and raises Cyclic adenosine monophosphate(cAMP) levels. As a second messenger, cAMP can activate PKA (231). PKA, via phosphorylated transcription factors like CREB and NFAT, regulates transcription of CH-related genes. PKA regulates the transcription of CH-related genes through phosphorylated transcription factors such as cAMP response element-binding protein (CREB) and NFAT (231).

Recent studies have found that ISO upregulates the expression of cardiac epidermal growth factor (Ereg) and nerve growth factor receptor (Ngfr). Knocking down Ereg downregulates the expression of Natriuretic Peptide Precursor B (Nppb) and Fibronectin 1 (Fn1), reduces cardiomyocyte size, and lowers fibronectin expression (232). Ngfr may promote the proliferation of cardiac fibroblasts and the synthesis of collagen by activating downstream signals such as p38 MAPK, leading to myocardial fibrosis and exacerbating the degree of CH (233). Neuraminidase 1 has also been found to interact with GATA4 to enhance Nppb expression, thereby promoting ISO-induced CH (234). It has been discovered that the activation of HDAC8/MMP12 stimulates Nppb expression and increases extracellular matrix degradation, thereby worsening CH (235). Additionally, SarcoEndoplasmic Reticulum Ca2+-ATPase (SERCA2a) is regarded as an important marker of pathological hypertrophy (236, 237). In a mouse HF model continuously stimulated by ISO for two weeks, the expression of SERCA2a in the heart was significantly reduced (238); The same down-regulation was also observed in neonatal rat cardiomyocytes when NE was applied for 24 h (239). Conversely, transfection of ascending aortic tract HF rats with adenovirus carrying the SERCA2a gene could significantly increase survival rates and restore the phosphocreatine/ATP ratio (240). Phospholamban (PLB), an endogenous inhibitor of SERCA2a, has an elevated expression that reduces the affinity of the calcium pump for Ca^2+^ and impays cardiac diastolic function (241). In ISO-induced exhaustion mice, the level of PLB significantly increased (242), while myocardial contractility was significantly enhanced after PLB knockout (243, 244).

#### 3.2.2 Cardiac fibrosis

Cardiac fibrosis (CF) is the excessive deposition of cardiac extracellular matrix (ECM) and fibrosis, causing structural and functional changes in the heart. It often occurs after myocardial injury or chronic inflammation (245). Many studies have indicated that NE and ISO can both induce CF (128, 246). For instance, in the cardiomyocytes and fibroblasts of rats treated with ISO, the expressions of basic fibroblast growth factor 2 (FGF2), collagen I and smooth muscle α-actin (α-SMA) significantly increased and promoted CF (247). However, CF development is extremely complex, involving various molecular mechanisms and signaling pathways, such as fibroblast activation and transformation, regulation by transforming growth factor β (TGF-β), inflammatory responses, and immune cell infiltration (248, 249).

In a healthy heart, fibroblasts are quiescent, primarily maintaining ECM homoeostasis by synthesizing and secreting small amounts of ECM components like collagen and fibronectin. When the heart is injured, fibroblasts are activated by factors such as TGF-β, Platelet-derived growth factor, and angiotensin II. This activation triggers downstream pathways, including the Smad and MAPK pathways, prompting fibroblast activation and their differentiation into myofibroblasts (MFB) (248). MFB enhance cellular contractility, exerting tension on myocardial tissue and affecting heart structure and function. They also overproduce and secrete ECM components, leading to excessive ECM deposition in the myocardial interstitial and gradual replacement of myocardial tissue with fibrosis (249). Other signal pathways, such as the Wnt/β-catenin and Notch pathways, are also involved in fibroblast activation and transformation (250, 251).

The SNS and the renin-angiotensin-aldosterone system (RAAS) form a tight “positive feedback” loop in heart diseases. The excitement of SNS can trigger the activation of RAAS, and the activation of RAAS in turn further intensifies SNS activities (244, 252). Central Angiotensin II enhances the excitability of preganglionic sympathetic neurons through the Angiotensin II Type 1 Receptor and increases the release of peripheral NE. The application of angiotensin-converting enzyme inhibitors (ACEI)/angiotensin II receptor blockers (ARB) can block this effect and reduce central sympathetic output (253). Meanwhile, the NE released by the renal sympathetic efferent fibers directly acts on the β1 receptor of parapylebular cells, stimulating the massive secretion of renin through the Gsα/cAMP/PKA signaling cascade, thereby initiating and amplifying the RAAS effect (254). RAAS activation is a key driving force for myocardial fibrosis. Angiotensin II and aldosterone induce the activation of myocardial fibroblasts, promote the synthesis of collagen and ECM (255, 256), and accelerate ECM remodeling by regulating the imbalance of matrix metalloproteinases (MMPs) and their inhibitors (TIMPs) (257, 258). In addition, the inflammatory response mediated by RAAS and the burst of reactive oxygen species (ROS) further aggravate CF and dysfunction. The above-mentioned mechanism reveals that the interactive dialogue between SNS and RAAS plays an important role in the process of CF (259, 260).

The persistent excessive activation of β-AR can cause cardiac pathological remodeling characterized by CF. For example, β-AR activation stimulates IL-18 secretion, promoting inflammation, and induces galectin-3 expression in macrophages, driving fibroblast to MFB transformation and causing CF (261, 262). Galectin-3 can mediate myocardial inflammation and promote CF through the TLR4/MyD88/NF- κB pathway (135). Blocking β-AR signaling will inhibit inflammasomes and improve CF (263). Studies have found that the activation of the β-AR-camp-PKA pathway triggers CF, which may be caused by promoting the expression of ROS, cardiomyocyte connective tissue growth factor, vascular endothelial growth factor, and TGF-β1 to trigger fibroblast proliferation (130, 264). Activating the NE-AR-PKC pathway upregulates BNIP3l expression, promoting cardiac fibroblast proliferation and ECM expression (265). Transient receptor potential (TRP) channels have also been found to regulate the proliferation, migration and differentiation of cardiac fibroblasts, as well as the synthesis and secretion of ECM (266). For instance, TGF- β1 activates TRPM7 channels to promote cardiac fibroblast proliferation, and TRPM7-mediated Ca^2+^ signaling enhances the fibrotic effects of TGF-β1 (267). Activation of TRPV4 can promote the proliferation and migration of fibroblasts (268). Recent studies have shown that methyltransferase-like 3, Insulin-like Growth Factor Binding Protein 3, and Set7 Methyltransferase are also involved in CF. Silencing METTL3 can down-regulate the expression of IGFBP3 and alleviate ISO-induced CF (269). Silencing Set7 also shows inhibition of CF (270).

## 4 Therapeutic strategy

Given the elevated risk of cardiac complications following AIS, active cardiovascular monitoring is imperative. Particular attention should be paid to patients with insular and right hemispheric ischemia, as these lesions may predispose to excessive activation of the SNS. AIS patients exhibit a high prevalence of electrocardiographic abnormalities, with study reporting incidence rates exceeding 90%, primarily manifesting as ST-segment elevation/depression, QTc prolongation, and AF (271). Meta-analyses have demonstrated significant elevations in BNP and NT-proBNP levels among AIS patients (272). Elevated NT-proBNP levels show strong correlations with ST-T segment alterations (273), and electrocardiographic evaluation may predict clinical outcomes in stroke patients (274). Furthermore, increased cardiac troponin levels are strongly associated with mortality risk in AIS (275), with elevated high-sensitivity cardiac troponin T (hs-cTnT) and troponin I serving as potential biomarkers of myocardial injury post-AIS (276, 277). These markers are also recognized as indicators of coronary artery disease risk (278). Notably, the National Institutes of Health Stroke Scale (NIHSS) score correlates with myocardial injury, as patients with NIHSS >10 demonstrate significantly higher troponin levels (279). In AIS patients with elevated hs-cTnT, focal fibrosis of the heart, left ventricular hypertrophy and left atrial dilation were observed using MRI (280). Subsequent echocardiographic evaluation is essential for assessing post-AIS cardiac dysfunction (281, 282), particularly reduced ejection fraction associated with systolic impairment (279). Therefore, systematic monitoring of electrocardiographic parameters (including ambulatory ECG), NIHSS scores, and cardiac biomarkers facilitates early identification of high-risk patients for cardiac sequelae, especially in those with SN-activating lesions such as insular or right hemispheric infarcts. When electrocardiographic abnormalities or biomarker elevations are detected, comprehensive cardiac functional assessment through echocardiography is strongly recommended. This multimodal approach enables timely intervention and improved management of stroke-associated cardiac complications.

Given that SNS overactivation and elevated catecholamine secretion may serve as key pathogenic drivers of neurogenic cardiac injury following AIS, targeting SNS hyperactivity and mitigating catecholamine toxicity may represent critical therapeutic strategies. Here, we focus on exploring the therapeutic potential of several pharmacological and technological interventions capable of suppressing SNS overactivation and reducing catecholamine toxicity in AIS-associated neurogenic cardiac injury. These include beta-blocker (BB), sodium-glucose cotransporter 2 inhibitors (SGLT2i), **angiotensin receptor-neprilysin inhibitor** (ARNI), and neuromodulatory techniques designed to attenuate SN tension.

BB can inhibit the binding of catecholamines (such as NE) to β-AR and reduce their cardiotoxic effects. Meanwhile, it also has multiple effects such as inhibiting SN excitation, improving ventricular remodeling and cardiac function (283, 284). A clinical study involving 5,212 ischemic stroke (IS) patients revealed that post-stroke BB administration was associated with reduced mortality and lower incidence of pneumonia (285). Recent *post-hoc* analysis of 5,049 AIS patients with baseline heart rates ≥100 bpm demonstrated significant long-term benefits of sustained BB therapy. Over a 10-year follow-up, discontinuation correlated with increased early mortality risk, whereas continuous BB use substantially decreased both all-cause mortality and stroke recurrence rates (286). Subgroup analysis identified enhanced therapeutic benefits in patients with elevated mean heart rates, concomitant atrial fibrillation (AF), or pre-existing coronary artery disease (286). These findings suggest that BB exert pronounced cardioprotection in tachycardic patients through dual mechanisms: heart rate reduction and suppression of pathological SN overactivation, collectively mitigating neurocardiac injury cascades.

A study found that for patients with AIS combined with high heart rate at admission, for every 10 beats per minute increase in heart rate, the relative risk of in-hospital death increased by 40% (287). Failure to receive BB treatment significantly increased the readmitted rate and mortality risk within 3 months and 1 year after discharge in elderly patients with HF combined with IS. Similarly, patients with a high heart rate also had a significantly increased related risk at 3 months or 1 year after discharge (288). Animal experiments found that metoprolol inhibits SNS excitation in MCAO mice, slowed down cardiac remodeling, and improved chronic cardiac dysfunction induced by SNS excitation (289). However, Eizenberg Y found that the use of beta-blockers before stroke was not associated with adverse functional outcomes or mortality 3 months after stroke (290), and Balla HZ also supported this conclusion through a meta-analysis (291). A clinical study involving 3,915 patients with IS also showed that BB treatment was not related to the functional prognosis and mortality of patients with IS complicated with hypertension (292). Although these studies have shown that the benefits of using BB treatment after AIS are not definite. However, they did not separately include patients with cardiac injuries such as high heart rate, AF or coronary heart disease after AIS in the analysis. This confounding might mask the actual efficacy of BB in specific populations. Regarding the impact of using BB when arrhythmia and cardiac complications (such as high heart rate, AF, HF, and coronary heart disease) occur after AIS, more high-quality studies are still needed for exploration at present.

SGLT2i have also been found to have an inhibitory effect on SN hyperactivity (293). Chiba et al. found that SGLT2 was expressed in both human and rat brains (294). SGLT2 was found to be distributed in the regions from the telencephalon, diencephalon to the brainstem (295). Interestingly, SGLT2 activation in the RVLM was associated with SN excitation (296, 297), and inhibition of SGLT reduced RVLM neuronal activity and suppresses SN output (298). Dapagliflozin was found to reduce the incidence of AF in patients with type 2 diabetes (299). Further meta-analysis revealed that SGLT2i reduced the risks of AF, atrial flutter and VT (300), but its protective effect on the posterior brain of AIS remains controversial (301). In addition, SGLT2i can improve HF by improving ventricular remodeling, modulating cardiac energy metabolism and ion exchange (302). Studies have found that SGLT2i can reduce sympathetic nerve activity through multiple mechanisms, among which regulating the feedback mechanism of the renal tubule-bulle apparatus is one of the key factors (303). SGLT2i reduces sodium reabsorption and increases sodium content in the distal convoluted tubules by inhibiting SGLT2 in the proximal convoluted tubules of the kidney (304). This activates the feedback mechanism of the renal tubule-parbulbar organ, causing the entry arterioles to contract and reducing the intraventricular pressure of the glomerulus. This mechanism not only improves the hemodynamics of the kidneys, but also indirectly reduces the activity of the sympathetic nervous system by decreasing renin secretion and lowering the activity of the renin-angiotensin system. By reducing sympathetic nerve activity, SGLT2i can decrease sympathetic nerve overload in the heart, alleviate myocardial injury and inflammatory responses. This mechanism is independent of its hypoglycemic effect and is effective for both diabetic and non-diabetic patients with HF (305). Therefore, SGLT2i is expected to become an effective drug for treating cardiac injury caused by excessive excitement of SNS after stroke.

The mechanism of action of ARNI is achieved by binding angiotensin II receptor antagonists (such as valsartan) and enkephalinase inhibitors (such as sacubitril). This combination drug can simultaneously inhibit the RAAS and enhance the activity of the natriuretic peptide system. The protective effect of sacubitril-valsartan, the representative drug of ARNI, in HF has been widely recognized (306). Meta-analysis shows that sacubitril-valsartan demonstrates superior efficacy in the treatment of heart failure patients after myocardial infarction compared with traditional ACEI and ARB. Specifically, it is manifested as a higher left ventricular ejection fraction, a lower left ventricular end-diastolic diameter and NT-proBNP level (307). In addition, for heart failure patients with reduced ejection fraction, sacubitril/valsartan also shows a lower all-cause mortality rate (308). This drug can also reduce the relative risks of cardiovascular death and HF hospitalization (309). This is related to the effects of RAAS inhibition, natriuretic peptide system activation, anti-inflammatory and antioxidant stress (310–312). Furthermore, sacubitril-valsartan has the effects of inhibiting the excitation of the SNS, reducing NE release and lowering arrhythmia. The mechanism is related to its regulation of the RAAS and natriuretic peptide systems (313). On the one hand, Sacubitril-valsartan reduces the excitability of the SNS and decreases the release of norepinephrine by decreasing renin secretion and inhibiting the activity of the RASS (314). On the other hand, it not only inhibits the SNS by inhibiting the degradation of natriuretic peptides (such as ANP and BNP), but also enhances the diuretic, diuretic and vasodilatory effects of natriuretic peptides, thereby reducing the burden on the heart (315). A study found that sacubitril-valsartan alleviated ISO-induced myocardial inflammation and fibrosis in rats and improved cardiac insufficiency (316). And therapeutic effect was related to the regulation of the TLR4/NF-κB and TGF-β1/Smad signaling pathways. The above results show that SGLT2i and sacubitril-valsartan can play a potential role in resisting the toxicity of catecholamines to the heart and inhibiting the excitation of the SNS, which provides possible therapeutic value for them in the treatment of neurogenic cardiac injury after AIS.

Several SN tension inhibition techniques, such as stellate ganglion block (SGB) and vagus nerve stimulation (VNS), hold promise as potential treatments for arrhythmias after AIS. SGB reduces SN excitability in the myocardium by blocking SN efferent fibers in the stellate ganglion, which in turn reduces cardiomyocyte autoregulation, triggered activity, and folding, leading to arrhythmia prevention and treatment (317). SGB has shown high therapeutic benefits and safety in the treatment of refractory angina and ventricular arrhythmias (318–320). Although SGB has many advantages, it still lacks high-quality clinical evidence to support it and has certain operational risks, difficulties in efficacy evaluation, large individual differences, and toxicity of local anesthetics. In the future, it is necessary to further optimize the operation techniques, improve the accuracy of the evaluation of the blocking effect, and carry out more high-quality randomized controlled trials. In recent years, VNS has received increasing attention in the field of arrhythmia treatment. Its mechanism of action lies in the fact that by stimulating the vagus nerve, it enhances the activity of the PN while inhibiting the overexcitation of the SN, which in turn improves the regulatory imbalance state of the cardiac AN and helps to restore normal cardiac rhythms and function (321). VNS reduces the infarct size, ventricular arrhythmia, and AF after myocardial ischemia/reperfusion and has the ability to improve the contractile function of the heart and ventricular remodeling (322). This may be related to the fact that VNS attenuated inflammation (323). Furthermore, the PN advantage induced by VNS is related to regulating the ANS in different regions of the cerebral cortex (324), which is of great significance for improving the dysregulation of AN after AIS. However, VNS surgery has a relatively high risk of long-term complications, such as arrhythmia, laryngeal hematoma, vocal cord injury, and breathing difficulties. In addition, it still faces problems such as inconsistent therapeutic effects and difficulty in standardizing stimulation parameters. In the future, it is necessary to optimize the stimulus parameters, reduce adverse reactions, and conduct more clinical trials to evaluate its safety and efficacy.

In conclusion, actively monitoring the indicators reflecting cardiac damage and focusing on patients with ischemic injury involving the right ANS may be of great value for the early identification of neurogenic cardiac damage after AIS. Suppressing SNS hyperactivity and mitigating catecholamine-mediated cardiotoxicity demonstrate therapeutic potential in ameliorating cardiac damage. These strategies should therefore be prioritized in clinical management to optimize neurocardiac outcomes.

## 5 Conclusion

While substantial advances have been made in understanding neurogenic cardiac injury after AIS, the regulatory mechanisms of the sympathetic-catecholaminergic axis within the brain-heart network under pathological conditions remain incompletely elucidated. Further investigation into how post-stroke sympathetic hyperactivity triggers myocardial injury–particularly through region-specific brain lesions and downstream catecholamine-mediated signaling pathways–remains a critical research priority with profound clinical implications for precision prevention and targeted therapies.

For the management of neurogenic heart disease caused by AIS, active monitoring of indicators reflecting cardiac damage should be carried out, with a focus on patients whose ischemic injury involves the right SNS. Regulating excessive excitation of ANS, reducing inflammation and oxidative stress may be the focus of preventing and treating myocardial injury after AIS. Future research should delve deeper into the mechanisms of toxicity of the sympathetic-catecholamine system on the heart after AIS. Efforts must be made to translate these theoretical insights into clinical practice and propel the development of clinical applications.
